# Epidemiology and Molecular Typing of *Trypanosoma cruzi* in Naturally-Infected Hound Dogs and Associated Triatomine Vectors in Texas, USA

**DOI:** 10.1371/journal.pntd.0005298

**Published:** 2017-01-17

**Authors:** Rachel Curtis-Robles, Karen F. Snowden, Brandon Dominguez, Lewis Dinges, Sandy Rodgers, Glennon Mays, Sarah A. Hamer

**Affiliations:** 1 Department of Veterinary Integrative Biosciences, Texas A&M University, College Station, Texas, United States of America; 2 Department of Veterinary Pathobiology, Texas A&M University, College Station, Texas, United States of America; 3 Department of Large Animal Clinical Sciences, Texas A&M University, College Station, Texas, United States of America; 4 Texas A&M Veterinary Medical Diagnostic Laboratory, College Station, Texas, United States of America; Universidad de Buenos Aires, ARGENTINA

## Abstract

**Background:**

*Trypanosoma cruzi* is the etiologic agent of Chagas disease throughout the Americas. Few population-level studies have examined the epidemiology of canine infection and strain types of *T*. *cruzi* that infect canines in the USA. We conducted a cross-sectional study of *T*. *cruzi* infection in working hound dogs in south central Texas, including analysis of triatomine vectors collected within kennel environments.

**Methodology/Principle Findings:**

Paired IFA and Chagas Stat-Pak serological testing showed an overall seroprevalence of 57.6% (n = 85), with significant variation across kennels. Dog age had a marginally significant effect on seropositivity, with one year of age increase associated with a 19.6% increase in odds of being seropositive (odds ratio 95% CI 0.996–1.435; p = 0.055). PCR analyses of blood revealed 17.4% of dogs harbored parasite DNA in their blood, including both seronegative and seropositive dogs. Molecular screening of organs from opportunistically sampled seropositive dogs revealed parasite DNA in heart, uterus, and mammary tissues. Strain-typing showed parasite discrete typing units (DTU) TcI and TcIV present in dog samples, including a co-occurrence of both DTUs in two individual dogs. Bloodmeal analysis of *Triatoma gerstaeckeri* and *Triatoma sanguisuga* insects collected from the kennels revealed exclusively dog DNA. Vector infection with *T*. *cruzi* was 80.6% (n = 36), in which *T*. *gerstaeckeri* disproportionately harbored TcI (p = 0.045) and *T*. *sanguisuga* disproportionately harbored TcIV (p = 0.029). Tracing infection status across dog litters showed some seropositive offspring of seronegative dams, suggesting infection of pups from local triatomine vectors rather than congenital transmission.

**Conclusions/Significance:**

Canine kennels are high-risk environments for *T*. *cruzi* transmission, in which dogs likely serve as the predominant parasite reservoir. Disease and death of working dogs from Chagas disease is associated with unmeasured yet undoubtedly significant financial consequences because working dogs are highly trained and highly valued.

## Author Summary

The parasite *Trypanosoma cruzi* can cause Chagas disease in humans and dogs. The parasite is typically spread through the feces of a blood-sucking ‘kissing bug’ insect. Despite many documented cases in dogs across Texas, there are few population-level research studies investigating canine Chagas disease in multi-dog kennels in Texas. We sampled a total of 86 dogs from three kennels in south central Texas. We found 58% of the dogs had antibodies indicating they had been exposed to the parasite, and 17% of the dogs had parasite DNA circulating in their blood. We found that over 80% of kissing bugs collected from the kennels were infected. Further, using a bloodmeal analysis technique, we detected that all tested bugs had recently fed on dog blood. Our findings indicate that dog kennels can serve as an environment where kissing bugs and dogs interact, and that outdoor, multi-dog kennels in areas with kissing bugs are high risk areas for *Trypanosoma cruzi* transmission in dogs.

## Introduction

Chagas disease in humans and dogs is caused by the hemoflagellate protozoan *Trypanosoma cruzi*. Active transmission cycles of the parasite occur across the southern USA, where infected triatomine ‘kissing bug’ vectors and wildlife reservoirs co-occur [1–4]. Canines in particular have been shown to be important reservoir and maintenance hosts throughout the Americas (see [5] for a comprehensive review). Although epidemiological studies of canine infection with *T*. *cruzi* in the southern USA are limited, cases are widespread, especially in Texas [4,6–11]. The first cases of canine *T*. *cruzi* infection in the USA were documented in Texas [9], and a recent retrospective study reported cases from across the state [8]. Studies have revealed anti-*T*. *cruzi* seroprevalences of 7.5% in stray dogs and 8.8% in shelter dogs across Texas [10,12]. However, given variation in clinical presentation in infected dogs, which ranges from asymptomatic to acute death or chronic heart disease [13], the veterinary implications of canine *T*. *cruzi* infections are uncertain. The absence of a canine vaccination or canine antiparasitic treatments against *T*. *cruzi* further complicates clinical case management.

Infection with *T*. *cruzi* can occur through the introduction of infected triatomine insect feces into skin lesions as the bug defecates on the host during or shortly after blood feeding. Oral transmission to dogs and wildlife may result from consumption of infected bugs or infected prey species [6,14,15]. Although congenital transmission in canines has been documented [16–18], the frequency with which this occurs is unknown. Accordingly, owners of seropositive breeding bitches are left with little information to guide breeding programs, except for the option of removal of positive females from breeding roles [19].

Although serologic testing is a common tool for diagnosing *T*. *cruzi* infections in dogs, parasitemia is known to peak as early as two weeks prior to detectable antibody levels. In dogs, experimental studies indicate that parasitemia occurs within days to four weeks after initial infection [20–23], with development of anti-*T*. *cruzi* antibodies detected at 15 days to 4 weeks post infection [23–25]. Further, *T*. *cruzi* genetic strain differences may play a role in disease outcomes in canines [21,25–27], with genetic variation occurring across geographic regions [28], yet there have been few investigations of which strains infect dogs in the USA [29]. Veterinarians and dog owners are faced with increasing diagnoses of canine *T*. *cruzi* infections, but a limited ability to understand the veterinary and public health consequences. Our objective was to compare multiple serological and molecular biology techniques to detect and characterize *T*. *cruzi* infections in a cross-sectional analysis of working hound dogs in a parasite-endemic region. We documented an active *T*. *cruzi* transmission cycle in kennels in south central Texas.

## Materials and Methods

### Ethics statement

Research use of all samples from dogs was secondary to collection for diagnostic purposes; the Texas A&M University Institutional Animal Care and Use Committee granted a formal waiver of ethical approval.

### Study design and sample collection

This study was motivated by unexplained deaths of several dogs from a large network of working hound dogs used for various scent detection functions, mainly across Texas. Several dogs died within a short time period, and postmortem histopathologic findings indicated that canine *T*. *cruzi* infection was the probable cause of the deaths. A representative histopathology report from a *T*. *cruzi*-seropositive six-year old female hound that died in August 2013 showed myocarditis and epicarditis—lesions consistent with chronic Chagas disease—although no protozoal amastigotes were observed in the myocardium or any other tissue examined (kidney, mediastinal lymph node, lung, liver, or spleen).

Using a cross-sectional study design, we assessed and sampled 86 working dogs from three multi-dog kennels in the network: 26 dogs from kennel A, 31 dogs from kennel B (where the sudden deaths and *T*. *cruzi* infection diagnosis had occurred), and 29 dogs from kennel C, which comprised all dogs in residence at these kennels. All dogs were Coonhounds, most were bred by the facilities, and ages ranged from approximately 6 months to 13 years. Dogs were housed in indoor-outdoor, open air, cement/concrete kennels located within a 50 km radius of each other in south central Texas counties. Canines had limited travel history, mainly within Texas.

General physical examinations (auscultation, rectal temperature, mucous membrane color, and generalized palpation) were conducted, and blood samples were collected between July and September, 2013. Over the following several months, opportunistic postmortem samples of blood and other tissues (heart, mammary gland, testicle, uterus) were collected from dogs euthanized for reasons unrelated to this study. Pedigree lineage records were analyzed to determine relationships among sampled dogs (i.e., dams and littermates). Triatomine bugs were opportunistically collected from kennels in the network by kennel staff and pest control operators in summer 2013.

### Serology

Serum aliquots were tested for anti-*T*. *cruzi* antibodies using indirect fluorescent antibody (IFA) testing at the Texas Veterinary Medical Diagnostic Laboratory (TVMDL; College Station, TX). All samples were screened for the presence of anti-*T*. *cruzi* antibodies at 1:20, 1:80 and 1:160 dilutions. According to TVMDL protocols, titer values of 20 or greater were considered positive for antibody.

The remaining serum was stored at -20°C until analyzed using the Chagas Stat-Pak chromatographic dipstick test (ChemBio, NY). The Chagas Stat-Pak test has previously been used for antibody-detection test in dogs [12,25,30], and may offer an economical alternative for rapid screening of population, as had been suggested of a similar rapid test [31]. Stored serum samples were tested according to manufacturer’s instructions and any development of a band at 15 minutes was considered positive for antibody. Band strength was noted as faint, medium, or bold. Samples positive using both IFA and Chagas Stat-Pak dipstick tests were considered seropositive in the calculation of population-level seroprevalence.

### Detection and characterization of *T. cruzi* DNA

An extraction kit (E.Z.N.A. Tissue DNA kit, Omega Bio-Tek, Norcross, GA) was used to extract DNA from 250 μL of clotted blood from dogs for which serology testing was also performed. Extracted DNA was analyzed using qPCR to detect parasite DNA.

Samples were first screened for presence of *T*. *cruzi* DNA using the real-time PCR Cruzi 1/Cruzi 2 primer set and Cruzi 3 probe [32,33]. This PCR amplifies a 166-bp region of a repetitive nuclear DNA sequence, and is sensitive and specific for *T*. *cruzi* when compared to other PCR techniques [34]. A Stratagene MxPro3000 instrument (Agilent Technologies, Santa Clara, CA) was used to amplify DNA under previously described thermocycling parameters [32], except with a 3-minute initial denaturation. Reactions consisted of 5 μL of template DNA, primers at a final concentration of 0.75 μM each, 0.25 μM of probe, and iTaq University Probes Supermix (BioRad Laboratories, Hercules, CA), in a total volume of 20 μL. Machine-calculated thresholds and reaction curves were visually checked to assure successful amplification. Samples producing cycle threshold (Ct) values of less than 34 were considered potential positives and subjected to further testing for confirmation and discrete typing unit (DTU)-typing.

A multiplex qPCR was used to confirm *T*. *cruzi* infection and determine *T*. *cruzi* DTU based on amplification of the nuclear spliced leader intergenic region (SL-IR) with the use of a panel of DTU-specific probes [35]. Reactions were 20 μL total volume using a QIAGEN Multiplex PCR Kit (QIAGEN, USA), run using the following protocol: 15 minutes at 95°C followed by 40 cycles of 95°C for 30 seconds and 60°C for 1 minute. Reactions were run on a BioRad CFX96 (Hercules, CA, USA). Both FAM and HEX dyes were used as previously described [35]; however, due to differing instrument capabilities, our reactions differed from published protocol [35] by substituting Cy5 and Tex615 dyes (Integrated DNA Technologies, Inc., Coralville, IA, USA) for Quasar670 and CAL Fluor Red610, respectively. Samples that yielded amplification curves on both the Cruzi 1/2/3 qPCR and the SL-IR qPCR were interpreted as PCR-positive in our analyses. Samples that fluoresced with FAM were classified as TcI, whereas samples that fluoresced with Tex615 were classified as TcIV; samples that fluoresced with both FAM and Tex615 were classified as mixed TcI/TcIV. Although different TcIII isolates have previously resulted in fluorescence of either Quasar670 alone or both Quasar670 and CAL Fluor Red610, the TcIV isolates previously tested were shown to only cause CAL Fluor Red610 fluorescence [35]. None of the samples we tested resulted in Quasar670 (here, Cy5) fluorescence. Supported by the subset that we definitively typed using TcSC5D gene sequencing (below), we classified samples with CAL Fluor Red610 (here, Tex615) fluorescence as TcIV.

In addition to probe-based DTU-typing, as an additional method to investigate strain-typing, a subset of samples were amplified using a primer set that amplifies a region of the TcSC5D gene, a putative lathosterol/episterol oxidase [36]. The 832-bp amplicons were visualized on 1.5% agarose gel with ethidium bromide, and sequenced using Sanger sequencing (Eton Bioscience Inc., San Diego, CA, USA). Geneious version 8 [http://www.geneious.com [37]] was used to visually review chromatographs and sequences, align forward and reverse sequences, and examine locations of key SNPs to determine DTUs [36].

Negative controls were included in each set of DNA extractions and PCR reactions. Positive controls included *T*. *cruzi* DNA extracted from a TcI isolate Sylvio X10 CL4 (ATCC 50800, American Type Culture Collection [ATCC], Manassas, VA, USA), an untyped isolate cultured from a published Texas canine case [38], a TcIV isolate from an infected Texas raccoon [39], and TcIV isolates from *T*. *sanguisuga* and *T*. *gerstaeckeri* from Texas.

### Risk factor analyses

Samples positive using both IFA and Chagas Stat-Pak dipstick tests were considered seropositive in the calculation of population-level seroprevalence. Blood samples classified positive by Cruzi 1/2/3 and SL-IR qPCRs were considered positive in calculation of population-prevalence of *T*. *cruzi* DNA in blood samples. To evaluate the relationship between potential risk factors and positive canines, bivariable analyses were performed using chi-squared, Fisher’s exact tests, or t-tests. Variables assessed were kennel (A, B, or C), age, and sex. Logistic mixed effect regression models were built using the lme4 package in Program R [40] to further investigate risk factors with p < 0.25 in the initial screening and risk factors with justification for inclusion based on published data. To determine the variation in positive dogs across age and sex, kennel was included as a random effect. To determine the variation in positive dogs across kennels, age was included as a random effect. Factors with values of p < 0.05 were considered significant. Odds ratios and 95% confidence intervals were calculated. Separate models were built for anti-*T*. *cruzi* antibody status and blood *T*. *cruzi* PCR status.

### Microscopic and molecular analysis of tissues

Tissues collected opportunistically from euthanized dogs were preserved in 10% neutral buffered formalin. Formalin-preserved samples were submitted for histopathologic examination with routine hematoxylin and eosin staining at the TVMDL and reviewed by a pathologist. Additionally, DNA was extracted from approximately 1 cm^3^ pieces of various fresh tissues using the same methods as the molecular processing of dog blood samples as described above. Given variation in parasite localization within tissues, up to five independent subsamples per tissue were tested.

### Characterization of vector *T. cruzi* infection and bloodmeal host identification

Bugs were identified to species using morphologic features [41]; sex and evidence of a recent bloodmeal were noted. After bugs were washed in 10% bleach solution and rinsed in distilled water, sterile instruments were used to dissect the bugs and isolate hindgut material. DNA was extracted from hindguts and tested for *T*. *cruzi* DNA and DTU determination using the same methods described above. Two-sample tests for equality of proportions with continuity corrections were used to compare the proportions of TcI and TcIV between infected *T*. *gerstaeckeri* and *T*. *sanguisuga*. In order to determine the source of recent bloodmeals, hindgut DNA was subjected to PCR amplification of host cytochrome B sequences using previously published primers and cycling conditions [42,43]. Reactions included 3 μL template DNA, primers at final concentrations of 0.66 μM each, and FailSafe PCR Enzyme Mix with PreMix E (Epicentre, Madison, WI) in a final reaction volume of 50 μL. Amplicons were visualized on 1.5% agarose gel with ethidium bromide, and sequenced using Sanger sequencing (Eton Bioscience Inc., San Diego, CA, USA). Resulting sequences were compared to existing sequences using Basic Local Alignment Search Tool (National Center for Biotechnology Information, US National Library of Medicine).

### Accession numbers

Sequences of the TcSC5D genetic region amplified from samples are available at NCBI GenBank; accession numbers are KX594832-KX594840

## Results

Blood samples from 86 dogs in three kennels were analyzed using a variety of serologic and molecular techniques to detect *T*. *cruzi* exposure and infection. Additionally, tissue samples were tested from 9 dogs. A total of 44 triatomine insects were recovered from the kennels for testing and analyses.

### Population data

General physical examinations (auscultation, rectal temperature, mucous membrane color, and generalized palpation) of the dogs at the time of sampling did not reveal any significant findings suggestive of clinical presentation of *T*. *cruzi* infection.

**Fig 1:**
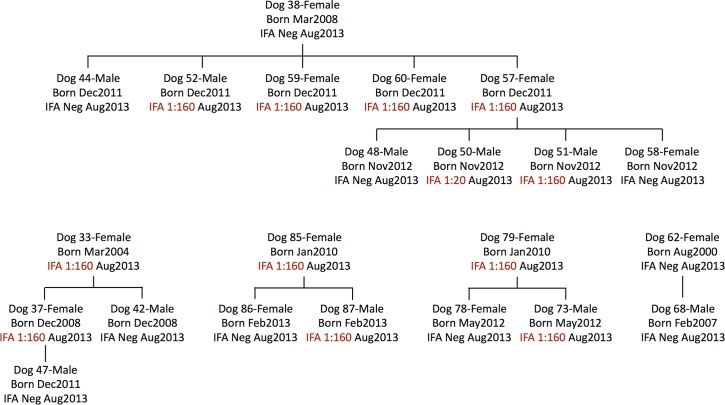
Lineages of five groups of related dogs. Each dog is represented by its number, sex, date of birth, and IFA status at time of August 2013 testing. Dams Dog 62, Dog 79, and Dog 85 were from kennel A, dams Dog 33, Dog 37, Dog 38, and Dog 57 were from kennel C.

The birthdate was known for 80 of the 86 dogs in the three kennels. Ages ranged from 6 months to 13 years, with a mean and median of 3.96 years and 3.58 years, respectively. Mean age and standard deviation at kennels A, B, and C was 4.05 ± 3.31 years (n = 26), 3.77 ± 2.64 years (n = 27), and 4.06 ± 2.68 years (n = 27), respectively. There were 15 dogs less than 1 year old (18.6% of 80). There were 39 males (45.3%) and 47 females (54.7%). At the time of the cross-sectional blood sampling, there were seven dams with a total of seventeen offspring that were included in the study (Fig 1).

### Serological results

A total of 56 of 86 (65.1%) dogs had an antibody titer value of 20 or greater on IFA, and 53 of 85 (62.4%) dogs were reactive on the Chagas Stat-Pak (Table 1). Combined, 49 of 85 dogs were positive on both antibody detection tests, yielding a seroprevalence of 57.6%. A single sample was positive on IFA with an antibody titer of 160, but was not tested on the Chagas Stat-Pak and was therefore not included in the overall seroprevalence estimate. There were 10 dogs positive on only one test and negative on the other; these dogs with discordant results were considered seronegative for the purpose of this study. Of these ten discordant samples: 4 dogs were negative on IFA but had faint (positive) lines on the Chagas Stat-Pak, and 6 dogs were negative on Chagas Stat-Pak but had IFA titer values of 20 (3 dogs), 80 (1 dog), and 160 (2 dogs). Overall seroprevalences at each kennel were: 46.2% at kennel A (n = 26), 71.0% at kennel B (n = 31), and 53.6% at kennel C (n = 28).

**Table 1:** Serological testing. Blood samples from working dogs were tested for anti-*T*. *cruzi* antibodies using IFA and Chagas Stat-Pak. Only those samples positive on both assays were considered positive for calculation of seroprevalence. *In addition to these 55 IFA-positive dogs, there was one additional dog with an antibody titer of 160 that was not run on the Chagas Stat-Pak.

		IFA	
		Positive	Negative	Total
**StatPak**	**Positive**	49	4	53
	**Negative**	6	26	32
	** Total**	55*	30	85

Analysis of canine serostatus in relation to lineage revealed both positive and negative littermates born to positive and negative dams. Four 20-month old pups were seropositive, despite the concurrently-tested dam being seronegative (Fig 1).

In addition to the main 86 dogs of this study, a litter of young pups was opportunistically sampled to gather data on serostatus and PCR status of neonatal pups born to a seropositive dam. A litter of six pups was born to a female who tested serologically positive (≥ 1:160) nine months previously, and these pups were serially sampled twice over 4 weeks. The dam accidentally smothered one of the pups one day after birth. Blood samples from that pup were PCR negative for parasite, although testing on Chagas Stat-Pak gave a faint (positive) band. The other five pups had blood sampled two weeks after birth and tested on Chagas Stat-Pak: two gave very faint (positive) bands and three were negative. At one month of age, all five were negative on Chagas Stat-Pak. None of the two week or one-month samples were PCR positive.

### Molecular detection and characterization of parasite DNA in blood samples

PCR analysis of 86 DNA extracts of blood clots revealed 15 (17.4%) positive samples. There were 23 samples with Ct values less than 34 on the initial Cruzi 1/2/3 qPCR and subjected to attempted amplification using the SL-IR PCR, of which 15 samples tested positive using the SL-IR PCR assay. Overall prevalences of PCR-positive dogs at each kennel were: 15.4% at kennel A (n = 26), 25.8% at kennel B (n = 31), and 10.3% at kennel C (n = 29).

Of the 15 PCR positive blood samples, the SL-IR assay revealed 9 TcI, 5 TcIV, and 1 TcI/TcIV mixed infections. Amplification and sequencing of the TcSC5D gene DNA target was successful in five blood samples. TcSC5D and SL-IR results were congruent, except one of the TcIV TcSC5D findings was characterized as a mixed TcI/TcIV by SL-IR PCR.

### Comparison of serology and PCR

Using the serological positivity criterion of being positive on both IFA and Stat-Pak assay, serology and PCR findings categorized 12 of 85 dogs (14.1%) as both seropositive and PCR positive (Table 2), 37 dogs (43.5%) as seropositive and PCR negative; 3 dogs (3.5%) as PCR positive and seronegative; and 33 dogs (38.8%) as both seronegative and PCR negative. One dog sample with a 160 IFA titer was not run on Chagas Stat-Pak; that dog was PCR negative. Two dogs that were PCR positive but did not meet the positivity criterion on both serological assays were positive on IFA with antibody titers of 20 and 80. Of the 37 seropositive, PCR-negative dogs, only one was less than 1-year old, indicating that seropositive dogs were not positive due to maternal antibodies.

**Table 2:** Results of serologic and PCR testing. Blood samples from working dogs were tested for anti-*Trypanosoma cruzi* antibodies using IFA and Chagas Stat-Pak; DNA extracted from the blood clots were tested for presence of *T*. *cruzi* DNA using two PCR assays. Samples were considered serologically positive if positive on both IFA and Chagas Stat-Pak and PCR positive if they were positive on both assays.

		Serology	
		Positive	Negative	Total
**PCR**	**Positive**	12	3	15
	**Negative**	37	33	70
	** Total**	49	36	85

### Risk factor analyses

Bivariable analysis of putative risk factors for canine seropositivity indicated that kennel (p = 0.146) and age (p = 0.077), but not sex (p = 0.535), were associated with p-values below the threshold significance level for inclusion in the regression model (Table 3). Bivariable analysis of putative risk factors for blood PCR-status indicated that neither kennel (p = 0.310), age (p = 0.344), nor sex (p = 0.863) were associated with p-values below the threshold significance level for inclusion in the regression model (Table 3). Nonetheless, all putative risk factors were retained in the regression models based on previous work [14,44–46]. In logistic regression models to predict serostatus while including kennel as a random effect, dog age was marginally positively (p = 0.055) associated with seropositivity, where one year of increase in canine age was associated with a 19.6% increase in the odds of being seropositive, and there was no effect of sex (p = 0.855) (Table 4). The odds of being seropositive were 6.6 (95% CI 1.32–32.88) times greater for dogs in kennel B than in the referent kennel (kennel A) (p = 0.022). In the logistic regression models to predict blood PCR status, none of the putative risk factors of kennel (p = 0.251 and p = 0.670), age (p = 0.296) or sex (p = 0.637) were significant.

**Table 3:** Bivariable analyses. Characteristics of working hounds dogs and serologic and infection status with *Trypanosoma cruzi*, Texas, 2013.

	Anti-*T*. *cruzi* antibody status			Blood PCR status		
Risk factor	Seropositive dogs (*n* = 49)	Seronegative dogs (*n* = 36)	p-value	PCR-positive dogs (*n* = 15)	PCR-negative dogs (*n* = 71)	p-value
Dogs in kennel, *n* (%)			0.146^a^			0.310^c^
A	12 (46.2)	14 (53.8)		4 (15.4)	22 (84.6)	
B	22 (71.0)	9 (40.9)		8 (25.8)	23 (74.1)	
C	15 (53.6)	13 (46.4)		3 (10.3)	26 (89.7)	
Age in years, mean (SE)^d^	4.4 (0.4)	3.2 (0.5)	0.077^b^	3.3 (0.8)	4.1 (0.3)	0.344^b^
Sex, *n* (%)			0.535^a^			0.863^a^
Female	29 (61.7)	18 (38.3)		9 (19.1)	38 (80.9)	
Male	20 (52.6)	18 (47.4)		6 (15.4)	33 (84.6)	

*n*: sample count; %: percentage; and SE: standard error.

^a^ Evaluated with Chi-squared.

^b^ Evaluated with t Student test.

^c^ Evaluated with Fisher exact test.

^d^ Ages of 6 dogs were unknown, and these dogs were not included in analysis.

**Table 4:** Regression models. Factors associated with *Trypanosoma cruzi* seropositivity and PCR-positivity in working hound dogs, Texas, 2013.

		Model to predict seropositivity			Model to predict PCR-positivity		
		OR	95% CI	p-value	OR	95% CI	p-value
Kennel	Kennel A	referent			referent		
	Kennel B	6.585	1.319–32.884	0.022	2.445	0.532–11.241	0.251
	Kennel C	1.376	0.332–5.702	0.660	0.683	0.119–3.939	0.670
	Age (years)	1.196	0.996–1.435	0.055	0.889	0.713–1.108	0.296
Sex	Female	referent			referent		
	Males	1.099	0.399–3.031	0.855	0.756	0.236–2.418	0.637

95% CI: 95% confidence interval.

### Microscopic and molecular analysis of tissues

A total of five tissue samples opportunistically collected from four IFA-positive dogs were examined histologically. Three of four cardiac samples had lesions consistent with chronic canine trypanosomiasis (Table 5), although no amastigotes were observed in any of the sections. Lesions included cardiomyofiber degeneration (ranging from minimal to moderate), with accumulations of lymphocytes, plasma cells, and rare macrophages. One uterine tissue section was viewed; no amastigotes or significant lesions were observed (Table 5).

**Table 5:** Opportunistic additional testing of serologically positive dogs. Serologic, molecular, and histologic results of tissue samples opportunistically collected from *T*. *cruzi*-infected dogs.

Dog ID	Sex	Age at time of sampling	Tissue tested and PCR results* (DTU detected)	Histopathology results
Dog 4	F	5 y	uterus—negative	NA
Dog 5	F	5 y	uterus—negative	NA
Dog 7	F	8 y	uterus—negative	NA
Dog 50	M	15 mo	• heart–positive (DTU TcIV) • testicle—negative	heart—minimal cardiomyofiber degeneration and loss with accumulation of a few lymphocytes and plasma cells
Dog 51	M	15 mo	• heart—negative • testicle—negative • blood—negative	heart—no significant lesions
Dog 53	M	3 y	• heart—negative • testicle—negative	NA
Dog 75	M	2 y	• heart–positive (DTU TcI) • testicle—negative	heart—multifocal, mild foci of cardiomyofiber degeneration and loss with interstitial fibrosis and accumulation of lymphocytes, plasma cells and rare macrophages
Dog 88	F	13 mo	• heart—positive (4/4) (DTU TcI) • blood—positive (2/5) • uterus—positive (4/5) (DTU TcIV) • mammary gland—positive (2/2) (DTU TcIV)	• heart–moderate cardiomyofiber degeneration and loss with accumulation of lymphocytes, plasma cells and a few macrophages • uterus—no significant lesions
Dog 432	M	6 mo	• heart—positive (DTU TcI) • blood—positive (DTU TcIV)	NA

*Fractions in parenthesis indicate number of positive subsamples over total subsamples tested.

*T*. *cruzi* DNA was detected in heart, blood, uterus, and mammary gland tissues collected opportunistically from multiple serologically-positive dogs (Table 5). Three dogs did not have detectable parasite DNA in tested uterine tissue, whereas four of the five samples from the body of the uterus of one dog were PCR positive. Three dogs did not have detectable parasite DNA in tested testicular tissue. One dog (Dog 88) had multiple parasite positive tissues, including heart, blood, uterus, and mammary gland. One dog (Dog 432) had PCR-positive blood and heart tissue.

Cardiac and uterine samples from one dog (Dog 88) revealed TcIV in uterine and mammary gland tissue and TcI in cardiac tissue; these results were congruent between SL-IR and TcSC5D DTU-typing methods. One dog (Dog 432) had TcI in heart tissue and TcIV in blood.

### Bugs

A total of 44 bugs (Table 6) were opportunistically collected in summer 2013 from the network of working dog kennels, including the three kennels that housed dogs tested in the cross-sectional serological study. Bugs included 16 adult *Triatoma gerstaeckeri* and 28 adult *T*. *sanguisuga*. Of the 36 insects that were tested for *T*. *cruzi*, 29 (80.6%) were positive, including 16 of 16 (100%) tested *T*. *gerstaeckeri* and 13 of 20 (65%) *T*. *sanguisuga*. The proportion of infected *T*. *gerstaeckeri* that harbored TcI was significantly greater than that of infected *T*. *sanguisuga* (*χ*^2^ = 4.026; p = 0.045), whereas the proportion of infected *T*. *sanguisuga* that harbored TcIV was significantly greater than that of *T*. *gerstaeckeri* (*χ*^2^ = 4.765; p = 0.029); Table 6). Based on visual examination, 30 of the 44 bugs had evidence of a recent bloodmeal in their guts. Of 24 bugs with sufficient bloodmeal volume for successful bloodmeal PCR and Sanger sequencing, all 24 had ≥97% identity to *Canis lupus familiaris* (domestic dog).

**Table 6:** Triatomine insects collected from kennels. *Triatoma* spp. bugs collected from kennels were tested for *Trypanosoma cruzi*. *T*. *cruzi* DTUs and bloodmeal sources were determined.

	Submitted	No. positive / no. tested (Infection prevalence)	*T*. *cruzi* DTUs	Bloodmeal source
*T*. *gerstaeckeri*	16	16/16	TcI (10 bugs; 62.5%)	*Canis lupus familiaris*
	(6M, 10F)	(100%)	TcIV (4 bugs; 25.0%)	(9/9 bugs)
			TcI/TcIV mix (2 bugs; 12.5%)	
*T*. *sanguisuga*	28	13/20	TcI (2 bugs; 15.4%)	*Canis lupus familiaris*
	(10M, 18F)	(65.0%)	TcIV (9 bugs; 69.2%)	(15/15 bugs)
			TcI/TcIV mix (2 bugs; 15.4%)	

## Discussion

Over half (57.6%) of a population of working hound dogs were seropositive for *T*. *cruzi*, and 17.4% harbored parasite DNA in their blood (Table 2). Additionally, we documented parasite DNA in heart, mammary, and uterine tissues in dogs from this network. A high (80.6%) infection prevalence was found in triatomines recovered from the kennels, and the only bloodmeal host detected in these bugs was dog. Coupled with the documented history of multiple deaths due to Chagas disease in these working dogs, our findings highlight a key role of dog kennels as a nidus of *T*. *cruzi* transmission. While Chagas disease impacts many types of dogs across the southern USA, including pet dogs and stray dogs, there is an additional unmeasured yet undoubtedly important financial consequence when Chagas disease impacts working dogs because they are highly trained and have a significant economic worth that results from the value of the duties they perform.

The 57.6% seroprevalence in these kenneled working dogs is much higher than the 8.8% seroprevalence found in a general population of dogs across Texas [12]. This difference is similar to findings in Louisiana, in which kenneled hunting dogs had a seroprevalence of 51.6%, which was higher than the 22.1% seroprevalence reflected in a general population of dogs in the surrounding area [30]. This population of working dogs we sampled was selected due to recent deaths in a focal kennel and is not representative of all kennels or south Texas. Dogs were considered at high risk to acquiring *T*. *cruzi* infection due to the presence of infected vectors in the kennel environment as well as outdoor working settings. All sampled dogs were intensively trained for pack tracking of lost and missing persons; when working, dogs trail intently while running and are undistracted by their surroundings. Therefore, although dogs may also encounter vectors outside the kennel environment, it is most plausible that infection was acquired within the kennels.

Older dogs were more likely to be seropositive, with approximately 19.6% increase in odds of seropositivity with each additional year of age, although the trend was only marginally significant (p = 0.055; Table 4). Increasing infection with age has been previously reported [7,14] and is expected, since older dogs have had longer opportunity to be exposed to *T*. *cruzi* and develop life-long seropositivity. Our findings of higher seroprevalence in older dogs are suggestive of an ongoing transmission cycle in these kennels, rather than an emerging recent phenomenon. It is unclear why dogs in kennel B were more likely to be seropositive than dogs in kennel A; one potential risk factor not examined in this study was additional outdoor kennels at kennel B that possibly served as refugia for triatomine bugs.

In this study, both serological diagnostic approaches (IFA and Chagas Stat-Pak) resulted in similar population-level estimates of seroprevalence (65.1% vs. 62.4%, (Table 1). However discordant results (positive and negative results on the same sample across different testing platforms) occurred in 10 dogs, the majority of which were negative on one test and only faintly positive (faint band or 20 endpoint titer) on the other. Although dogs with discordant results were interpreted as seronegative in our study, at least some of these dogs were infected, based on PCR-positive results in 2 dogs, likely reflecting acute infections. This observation underscores the importance of using personal protective equipment when handling canine blood even from seronegative individuals.

While the Stat-Pak has not been validated using dogs with known infection histories, this test has shown high sensitivity (87.2–100%) and specificity (93.2–98.6%) in human samples when compared with other serological techniques [47–49]; however, others have found considerable variation and lower sensitivity (26.6–87.5%) [50]. It is difficult to compare canine infection prevalence across studies because data from the same diagnostic tools may be interpreted differently. For example, whereas we interpreted any development of color to indicate a positive result for the Stat-Pak as per manufacturer’s instructions, others have considered faint band development as negative [30]. Further, in our study, serum dilutions for IFA of 1:20 or higher were considered positive for antibody as per TVMDL reporting standards. In other canine *T*. *cruzi* studies, however, dilutions interpreted as positive included those equal to or greater than 1:128 or 1:160 [4,8] (however, see [51]); and one previous study found chronically infected dogs produced positive results of serum dilutions ranging from 1:120 to 1:320 [20]. Faint bands on the Stat-Pak, low antibody titers on the IFA, and discordant results across multiple testing platforms may result from *T*. *cruzi* strain type variation, weak immune response, an early, rising antibody response to a recent infection, and variation in test sensitivity or specificity. Imperfect diagnostics and the absence of a gold standard indeed represent one of the major challenges in canine Chagas disease research.

Prior research of experimental chronic Chagas disease in dogs has demonstrated that multiple extractions and PCRs are needed to ensure detection of *T*. *cruzi* DNA from whole blood samples [52], although another study found that *T*. *cruzi* DNA was more likely to be detected the blood clot (which was used in this study) than buffy coat or whole blood samples [53]. One study conducting controlled experimental reinfection in dogs found that parasitemia was not as common in reinfections as it was in initial infection, and that parasitemia profile varied depending upon the individual dog [23]. While PCR of samples does not confirm the presence of whole, viable parasites in the blood, findings of parasite DNA in the blood suggest that positive dogs could potentially be infectious to blood-feeding insect vectors. It is likely the relatively high prevalence of *T*. *cruzi* DNA found in this study (17.4%) reflects the timing of the blood sampling (late July) corresponding with the time of year kissing bugs are most likely to be encountered in Texas [54]. With potential for continued exposure to kissing bugs and repeat infections with *T*. *cruzi*, it is possible that dogs with positive serological and positive PCR results (Table 2) could have been recently reinfected. Additional diagnostic difficulties are the result of parasitemia waning after initial infection [55] or lower parasitemic peaks from reinfection [23].

In comparing molecular and histology results, we found that all four PCR-positive hearts subjected to histology were associated with lesions consistent with chronic *T*. *cruzi* infection, although no amastigotes were seen in heart samples (Table 5). The lack of apparent amastigotes is not surprising, however, given that experimental studies have shown parasites are not always histologically detected in cardiac tissue of chronically infected dogs [20,23]. Further, *T*. *cruzi* strain type can also influence level of cardiac damage and presence of amastigotes [22], although more research is needed on pathology variation owing to parasite strain.

There is an interest, particularly in the canine breeding community, in whether *T*. *cruzi* can be sexually transmitted between dogs. We used PCR testing to evaluate testicle and uterine samples from seropositive dogs. None of four testicle samples were positive, but small sample size and conflicting reports in previous literature [56–58] leave us unable to draw firm conclusions. Of four uteri tested, we detected a single positive uterus in which four of the five samples taken from the body of the uterus were positive. The mix of positive and negative samples suggests that *T*. *cruzi* distribution in the tissue is not uniform. The detection of parasite DNA in uterine tissue supports previous reports of transplacental transmission of this parasite in dogs and in humans [17,59]. In addition to reproductive tissues, the potential for transmammary transmission has been suggested by others [14,16]. Our finding of *T*. *cruzi* DNA in mammary gland tissue was in a dog that also had evidence of parasites in heart and uterine tissue.

Congenital transmission of *T*. *cruzi* in dogs has been shown, with one study finding circulating antibodies in 45-day old pups born to experimentally infected dams [17], and is a concern to breeders. We conducted a cross-sectional study with concurrent sampling of dams and their offspring, and the infection status of dams was not specifically known at the time of whelping. Although this study design limits the ability to draw conclusions about congenital transmission in this setting, the observed patterns of infection across family lines are useful for inferring transmission pathways. For example, we found both seropositive and seronegative littermates from a seronegative dam, supporting the likelihood of local vector-borne transmission. In contrast, we observed several seropositive bitches associated with both seropositive and seronegative offspring; scenarios for which congenital transmission cannot be rule out. None of the PCR-positive dogs in this study were the offspring/dams of any other concurrently-sampled PCR-positive dogs. Additionally, although no parasite DNA was detected in six young pups from a seropositive dam, faint bands produced on the Chagas Stat-Pak test on early blood draws might be the result of maternal antibodies circulating in the pups. Additional research is needed regarding congenital transmission rates and relation between maternal and self-produced anti-*T*. *cruzi* antibodies.

We found higher infection prevalence (>80%) in kissing bugs than recent statewide estimates of 63% and 51% [54,60]. Bloodmeal analysis of the triatomines revealed all evaluated bugs had fed on dogs (Table 6). Other studies in the USA have found evidence of dog blood in triatomines, including bugs associated with houses and dog kennels [61–64]. High infection prevalence in vectors collected from canine quarters, combined with evidence of bugs feeding on dogs, supports vector-host contact and parasite transmission to dogs. Combined with our findings of PCR positive dog blood, it is likely that dogs are the source of vector infection and serve as the main reservoir in this setting. Prevention of canine infection with *T*. *cruzi* relies heavily on vector control. Integrated pest management strategies consisting of pesticide use, barrier methods (netting or mesh around kennels), and physical management of dogs (moving dogs to indoor facilities at night) have been employed in different areas around Texas.

We found TcI and TcIV infections in dogs, including three dogs infected with both DTUs. These dogs may have been re-infected given that the vector populations in the same areas also harbored both strains (Table 6). Previous strain typing efforts of limited dog samples from the USA have shown almost exclusively TcIV infections [29,65], although one TcI/TcIV mixed infection was documented in a USA dog [29], and mixed strain infections have been documented in dogs in Columbia [66]. *T*. *gerstaeckeri* were disproportionately infected with TcI (p = 0.045), whereas *T*. *sanguisuga* were disproportionately infected with TcIV (p = 0.029), in contrast to previous findings of only TcI in limited *T*. *sanguisuga* samples from the eastern USA [29]. Differing host preferences and geographic distribution of these triatomine species [60] may put geographically disparate dog populations at risk of acquiring different strains of *T*. *cruzi*. Previous research suggests that parasitemia, antibody development, and disease may vary according to strain type of *T*. *cruzi*, as well as length of infection and infected host species [21,22,67,68]. We found DTU determination using the probe-based qPCR [35] was more useful than the TcSC5D gene target assay, likely because the latter assay was developed using DNA from pure parasite culture [36] was not optimized for use in field-collected samples with mixed DNA populations [see [39,69]].

Although dogs have been shown to be important *T*. *cruzi* reservoirs in areas of Latin America [66,70,71], with one model of a rural Brazilian village estimating that an infected dog could infect one triatomine per day [72], the ecological settings of dogs in central Texas may limit their importance as reservoirs of human infections. Dogs in central Texas are typically housed either in a kennel separate and somewhat distant from the human dwelling or indoors in a house constructed with screens and doors that limit bug entry. The infected Texas dogs likely serve as reservoirs within the kennel setting, serving to infect bugs that can subsequently infect other dogs. However, the infectiousness of dogs to bugs has been shown to vary widely, and depends upon a variety of factors, including: dog body condition, coinfections, dog history of infection, vector competence, bug bloodmeal size, and bug feeding duration ([73], see [5] for a comprehensive review). Given our findings of high infection prevalence in dogs and vectors that fed on dogs, we conclude canine kennels represent a high-risk environment for *T*. *cruzi* transmission.
